# Kinetic Analysis Demonstrates a Requirement for the Rat1 Exonuclease in Cotranscriptional Pre-rRNA Cleavage

**DOI:** 10.1371/journal.pone.0085703

**Published:** 2014-02-03

**Authors:** Konstantin Axt, Sarah L. French, Ann L. Beyer, David Tollervey

**Affiliations:** 1 Wellcome Trust Centre for Cell Biology, University of Edinburgh, Edinburgh, Scotland; 2 Department of Microbiology, Immunology and Cancer Biology, University of Virginia Health System, Charlottesville, Virginia, United States of America; Rutgers New Jersey Medical School, United States of America

## Abstract

During yeast ribosome synthesis, three early cleavages generate the 20S precursor to the 18S rRNA component of the 40S subunits. These cleavages can occur either on the nascent transcript (nascent transcript cleavage; NTC) or on the 35S pre-rRNA that has been fully transcribed and released from the rDNA (released transcript cleavage; RTC). These alternative pathways cannot be assessed by conventional RNA analyses, since the pre-rRNA products of NTC and RTC are identical. They can, however, be distinguished kinetically by metabolic labeling and quantified by modeling of the kinetic data. The aim of this work was to use these approaches as a practical tool to identify factors that mediate the decision between utilization of NTC and RTC. The maturation pathways of the 40S and 60S ribosomal subunits are largely distinct. However, depletion of some early-acting 60S synthesis factors, including the 5′-exonuclease Rat1, leads to accumulation of the 35S pre-rRNA and delayed 20S pre-rRNA synthesis. We speculated that this might reflect the loss of NTC. Rat1 acts catalytically in 5.8S and 25S rRNA processing but binds to the pre-rRNA prior to these activities. Kinetic data for strains depleted of Rat1 match well with the modeled effects of strongly reduced NTC. This was confirmed by EM visualization of “Miller” chromatin spreads of nascent pre-rRNA transcripts. Modeling further indicates that NTC takes place in a limited time window, when the polymerase has transcribed ∼1.5Kb past the A2 cleavage site. We speculate that assembly of early-acting 60S synthesis factors is monitored as a quality control system prior to NTC.

## Introduction

**Figure 1:**
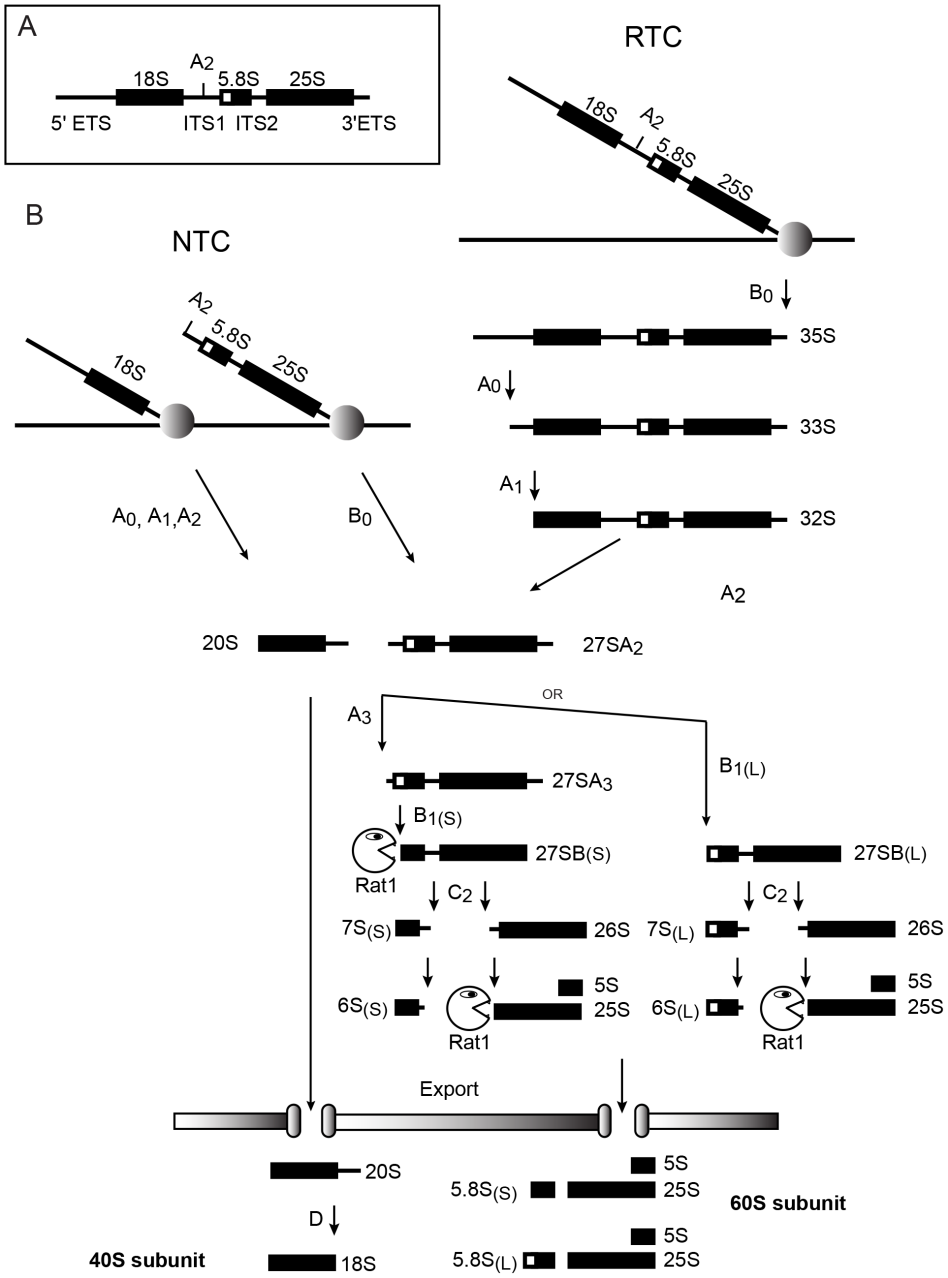
Pre-rRNA processing pathways in budding yeast. (A) Structure of the 35S pre-rRNA, showing the location of cleavage site A2. (B) Pre-rRNA processing pathways via nascent transcript cleavage (NTC) and released transcript cleavage (RTC). The points at which Rat1 functions as a 5′ exonuclease are indicated.

During the yeast ribosome synthesis pathway, the 18S, 5.8S and 25S rRNAs are cotranscribed as a single precursor that undergoes a multi-step processing pathway to generate the mature rRNAs (Figure 1). Three endonuclease cleavages generate the 20S pre-rRNA, which is matured into the 18S rRNA component of the 40S subunit. Subsequently, the mature 5.8S and 25S rRNAs of the 60S subunit are generated by a combination of endonuclease cleavages followed by exonuclease digestion. The first committed step on the major 60S synthesis pathway is endonuclease cleavage at site A3 by the RNA-protein complex RNase MRP [1]–[3]. Site A3 then acts as an entry point for the 5′-exonucleases Rat1 and Rrp17, which degrade the pre-rRNA back to site B1(S), the 5′ end of the major form of the 5.8S rRNA [3]–[5].

The cleavages at sites A0-A2 that release the 20S pre-rRNA can take place either on the nascent pre-rRNA transcripts during transcription (termed nascent transcript cleavage: NTC) or following transcription termination and release of the 35S pre-rRNA (termed released transcript cleavage; RTC) (Figure 1B). Since the products of RTC and NTC have the same sequences, they cannot readily be distinguished by “conventional” RNA analyses, such as northern hybridization, primer extension or RT-PCR. NTC can, however, be assed by electron microscopy (EM) of nascent transcripts in “Miller” chromatin spreads [6], which allows the cleaved, nascent pre-rRNA to be visualized. The NTC:RTC ratio can also be determined by fast kinetic analyses of the appearance of labeled, newly-synthesized pre-rRNA [7]. This relies on the fact that newly labeled 20S region of the pre-rRNA can only be observed as a discrete species when cleavage at sites A1 and A2 has occurred. Cleavage at these sites is tightly coupled, and the 20S is liberated more rapidly by NTC than RTC. In RTC polymerase must travel to the 3′ end of the 35S pre-rRNA, located more than 4kb downstream from site A2, before cleavage occurs. In contrast, NTC allows cleavage and 20S appearance before the polymerase has reached the end of the transcription unit. This time difference can be detected by metabolic labeling, following the incorporation of [^3^H] uracil into pre-rRNA species [7]. The existence of NTC can be deduced by inspection of labeling data, however, determining the efficiency of cleavage requires mathematic modeling of the experimental data. For this, a model was developed that used the existing knowledge of the pre-rRNA species and their inter-conversion to predict the sum of label incorporation into each pre-rRNA during the approach to steady state. The predicted curves could then be compared to the experimental data to assess whether the parameters chosen were appropriate. The initial model was implemented in MS Excel [7], which has the advantage of being widely used and understood by researchers. However, there are significant limitations to the use of MS Excel, particularly in the time intervals that can be modeled and the automated curve fitting available. Moreover, the model relied on lookup tables that were manually generated with obvious potential for errors. To address these weaknesses we rewrote the model in Mathematica and used this version for the analyses described here.

The composition of pre-ribosomal complexes assembled on the nascent transcripts remains poorly characterized. Specifically, there are no available data on the relative importance of specific 90S or pre-40S components for NTC versus RTC. However, numerous 90S and pre-40S maturation factors are apparently required for all 20S pre-rRNA synthesis, indicating that the actual pre-rRNA cleavage factors for NTC and RTC largely overlap. Following separation of the precursors to the 40S and 60S subunits by cleavage at site A2, the two pathways were believed to be entirely separate. This reflected initial analyses of yeast ribosome synthesis factors, which revealed that depletion of most factors blocked production of either the 40S or 60S subunit, with only a very few factors required for production of both subunits. However, a substantial group of proteins was subsequently identified that appeared to have anomalous behavior (see, for example [8]–[10]). Depletion of these factors blocked 5.8S and 25S synthesis but did not prevent 18S production, indicating that they are *bona fide* 60S synthesis factors. However, their depletion or mutation substantially perturbed the 18S maturation pathway, with elevated levels of the 35S pre-rRNA and appearance of the 23S RNA. The 23S RNA is produced by cleavage at site A3 in the absence of prior cleavage at sites A0, A1 and A2 (Figure 1B), and is generally regarded as an aberrant, non-productive processing intermediate since it has long been observed in strains that show impaired 18S synthesis [11] and is a known target for the TRAMP and exosome RNA surveillance factors [12], [13]. This situation appeared counterintuitive since the loss of the early processing factors on the 18S synthesis pathway did not clearly impact on subsequent 60S maturation, whereas loss of 60S synthesis factors had a negative effect on earlier steps in the pathway. The 60S synthesis factors showing such behavior included the Rat1 exonuclease, which seemed unlikely to participate directly in the endonuclease cleavage at sites A0-A2. The delay in A0-A2 cleavage was initially proposed to reflect a requirement for the assembly of much or all of the ribosome synthesis machinery with the pre-rRNA prior to the initiation of processing, perhaps as a quality control step [14]. However, the development of proteomic techniques for the analysis of yeast pre-ribosomes demonstrated that this is not the case, since pre-40S complexes contain few 60S subunit synthesis factors [15], [16].

These observations suggested the possibility that the observed effects of early 60S synthesis factors on 35S and 20S processing might actually reflect the specific loss of cotranscriptional cleavage. Analyses of the timing of the association of Rat1 with pre-ribosomes indicated that it is bound prior to cleavage at site A3 [17], suggesting that it might be present in particles on the nascent transcripts at the time of NTC at sites A0-A2. We therefore used kinetic labeling supported by mathematical modeling and EM analyses to address the requirement for Rat1 in cotranscriptional pre-rRNA cleavage.

## Materials and Methods

### Experimental Methods

#### Yeast strains, growth and labeling

Strains used were derived from W303-1a, with *P_MET3_::RAT1* integrated at the *RAT1* locus and carrying a *URA3* plasmid (pRS316) to allow growth in medium lacking uracil. Plasmids used are listed in Table 1. The effects of Rat1 depletion were analyzed in this strain additionally transformed with pRS315 (empty plasmid; strain YEAH212), pRS315-*RAT1-HA* (expressing functional HA-tagged Rat1; strain YEAH213) or pRS315-*rat1(D235A)-HA* (expressing catalytically inactive, HA tagged Rat1_D235A_; strain YEAH214).

**Table 1:** Plasmids used in this project.

Plasmid Name	Comments	Reference
pRS316	Contains *URA3* sequence	
pRS315	Empty plasmid	[21]
pRS315-*RAT1-HA*	Expresses functional Rat1	[21]
pRS315-*rat1(D235A)-HA*	Expresses catalytically inactiveRat1_D235A_	[21]

Following addition of methionine for 8 h, the Rat1-depleted and complemented strains were pulse-labeled with [^3^H-5,6] uracil. Cells were harvested at 30 sec intervals by transfer of 900 µl culture samples into 900 µl ethanol at −80°C, to rapidly inhibit label uptake and RNA metabolism. RNA was extracted, separated on denaturing agarose/glyoxal gels and transferred to nylon membranes (Hybond N+). RNA labeled with [^3^H] uracil was visualized using a Fuji imager (Figure S1). To allow different data sets to be directly compared, signals were normalized to the average values for the 27SA pre-rRNA plateau, which was previously shown to give the most reliable results [7].

#### Northern hybridization

Following determination of the tritium signal, the filters were subsequently hybridized with [^32^P] labeled probes directed against the mature 18S and 25S rRNAs, to correct for differences in extraction and RNA recovery. Oligonucleotide probes used were CATGGCTTAATCTTTGAGAC for 18S and CTCCGCTTATTGATATGC for 25S.

#### Chromatin spreads

Yeast cell cultures were grown in SCglu medium containing 1 M sorbitol, and lacking leucine and methionine to OD_600_ ∼0.09.Cultures were split into 2 flasks, with methionine (5 mM final concentration) added to one of the flasks. After 5 h, 1 ml volumes of the cultures were harvested and prepared for Miller chromatin spreading as previously described [18]. At the time of harvest, the OD_600_ of the cultures was between 0.4–0.6.

The spreading experiment was done independently four times, with multiple EM grids prepared for each strain each time. All grids were thoroughly scanned and all active rRNA genes were photographed. All genes in which the structure of the nascent transcripts could clearly be seen were analyzed for the presence or absence of nascent transcript cleavage. Sample sizes in the presence of methionine were 115 genes (Rat1 plasmid strain), 96 genes (empty plasmid strain), and 78 genes (Rat1-_D235A_ plasmid strain).

### Computational Methods

#### Mathematical modeling

An MS Excel based mathematical model of pre-rRNA metabolism [7] was transferred to Mathematica 6.0 (Wolfram Research Company) with the aim of making the model more portable and potentially allowing automatic parameter. The standard procedure was to import MS Excel sheets containing raw pre-rRNA time-course data and to process the data in Mathematica 6.0 with a global optimization application (S Fit), or to compare model and experimental data with a manual curve fitting program (M Fit). The source codes of the programs are available from the authors. Each program was validated by comparing results to the original MS Excel sheet containing the published model [7]. All model responses were normalized to the average 27SA model response. This facilitated comparison to the experimental data, which was also normalized to 27SA average intensity. Optimized parameters are listed in Tables 2 and 3.

**Table 2:** Parameters used for modeled curves shown in Figure 2.

Parameter	70% NTC	30% NTC
V	15nt sec^−1^	15nt sec^−1^
P	0.7	0.3
Equilibration time	45	45
Lifetime 35S	10	10
Lifetime 20S NTC	110	110
Lifetime 20S RTC	110	110
NTC window	1000nt	1000nt
Lifetime 27SA NTC	35	35
Lifetime 27SA RTC	95	95
Lifetime 27SB NTC	45	45
Lifetime 27SB RTC	45	45
Processing time 27SA->B	1	1

V: Velocity of transcription in nucleotides (nt) incorporated sec^−1^.

P: Probability that pre-rRNA will undergo nascent transcript cleavage (NTC) rather than released transcript cleavage (RTC).

NTC window: The distance traveled (in nt) by the transcribing polymerase downstream of site A2 prior to the NTC event.

Equilibration time: Time required for [^3^H] tritium uptake and equilibration of the internal nucleotide pool, prior to linear incorporation of label into newly synthesized RNA.

Lifetimes are in seconds.

**Table 3:** Parameters used for modeled curves shown in Figure 4.

Parameter	Rat1-expressing	Rat1-depleted
V	30nt sec^−1^	20nt sec^−1^
P	0.7	0.3
Equilibration time	45	45
Lifetime 35S	9	30
Lifetime 20S NTC	180	160
Lifetime 20S RTC	180	160
NTC window	1500nt	1500nt
Lifetime 27SA NTC	35	45
Lifetime 27SA RTC	85	100
Lifetime 27SB NTC	80	80
Lifetime 27SB RTC	80	80
Processing time 27SA->B	1	60

Terms are as in Table 2.

In fitting the curves, the lifetime 35S was a fixed parameter in the sense that its minimum lifetime was set to a value (10 sec) derived from our previous work, and reported in the literature. Similarly, the NTC window was set to 1500 nt. The transcription speed was then set according to the measured accumulation curve for 35S. The transcription time equals the time from the first inflection (start of labeling) to the second inflection of the 35S curve (steady-state). This value was entered to reduce the number of variables the program needs to go through and therefore processing time.

#### Statistical methods

Following the EM analyses, a Chi-squared test was used to determine if the proportion of genes with no NTC was significantly altered by the experimental conditions. This test was used to determine whether the null hypothesis, that the two samples are not different, could be rejected. The test was also applied to the individual strains in the absence versus the presence of methionine. The fraction of genes without NTC showed no significant change in the absence or presence of methionine in the strain expressing the Rat1 plasmid (P = 0.6), but increased significantly in the presence versus absence of methionine in the strain with the empty vector and in the strain expressing the Rat1_ D235A_ catalytic mutant (P values <10^−30^).

#### Global parameter optimization

Manual fitting of every individual parameter is time consuming and does not assure that the optimal solution has been found. To solve this problem an automatically conducted fitting would be advantageous. A global fitting program was written in Mathematica to perform this task and was named S fit. The following basic algorithm was used to find best fit values for model parameters(19).
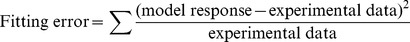



Experimental data were subtracted from the results obtained when the model was populated with a random set of parameters. This procedure was continued until the smallest possible difference between experimental data and model outcome was reached (for a defined set of parameters). The squared difference between the experimental data and the model response divided by experimental data is also a measure for the quality of the fit. The better the fit the lower this number will be. Best-fits can be attained, but limits must be set in order to obtain parameters that make biological sense. It was important to optimize parameters for several pre-rRNA species at once to allow fitting. For example, the lifetime of 35S RNA and the probability of NTC (P) are interdependent variables and thus it is not possible to obtain a single best-fit using only these data as input. The surface graph in Figure S1 clearly shows this from different perspectives. This problem can be circumvented by fixing one parameter (lifetime 35S or P) based on prior experimental data, or by including models for additional species e.g. 20S and 27SA in the automatic fitting process. Figure S2 shows a surface graph where parameter P was fixed (at 70%) and only the lifetime 35S was set free. There is a clear valley at 15 seconds, which would represent the optimum fit (using only the 35S model). When doing optimizations, S Fit considers all models in the fitting. The search algorithm is based on a least squares formula (below). The best fit was constrained by setting minimum lifetimes, in some cases maximum lifetimes and ranges for the NTC window.
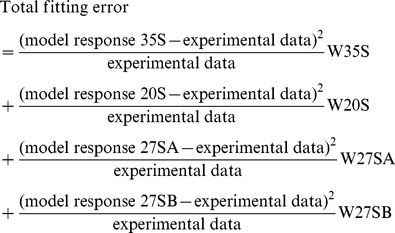



This is the formula by which the calculation of the fitting error was performed. The total fitting error was minimized by the search algorithms in the Mathematica package.

## Results

### Predicting the Effects of Changes in Cotranscriptional Processing Efficiency on the Kinetics of Pre-rRNA Labeling

A model for pre-rRNA processing was previously reported [7] to allow the relative frequency of NTC and RTC to be determined from in vivo labeling data. The labeling used was [^3^H-5,6] uracil, which was added to exponentially growing cultures without chase. The unlabeled pre-rRNAs in the cells are at steady-state, and the metabolic labeling reveals the kinetics of the approach to this steady state over time [7]. To analyze the data we developed a model in Mathematica 6.0 (Wolfram Research Company) for the time course of label incorporation into the pre-rRNAs.

**Figure 2:**
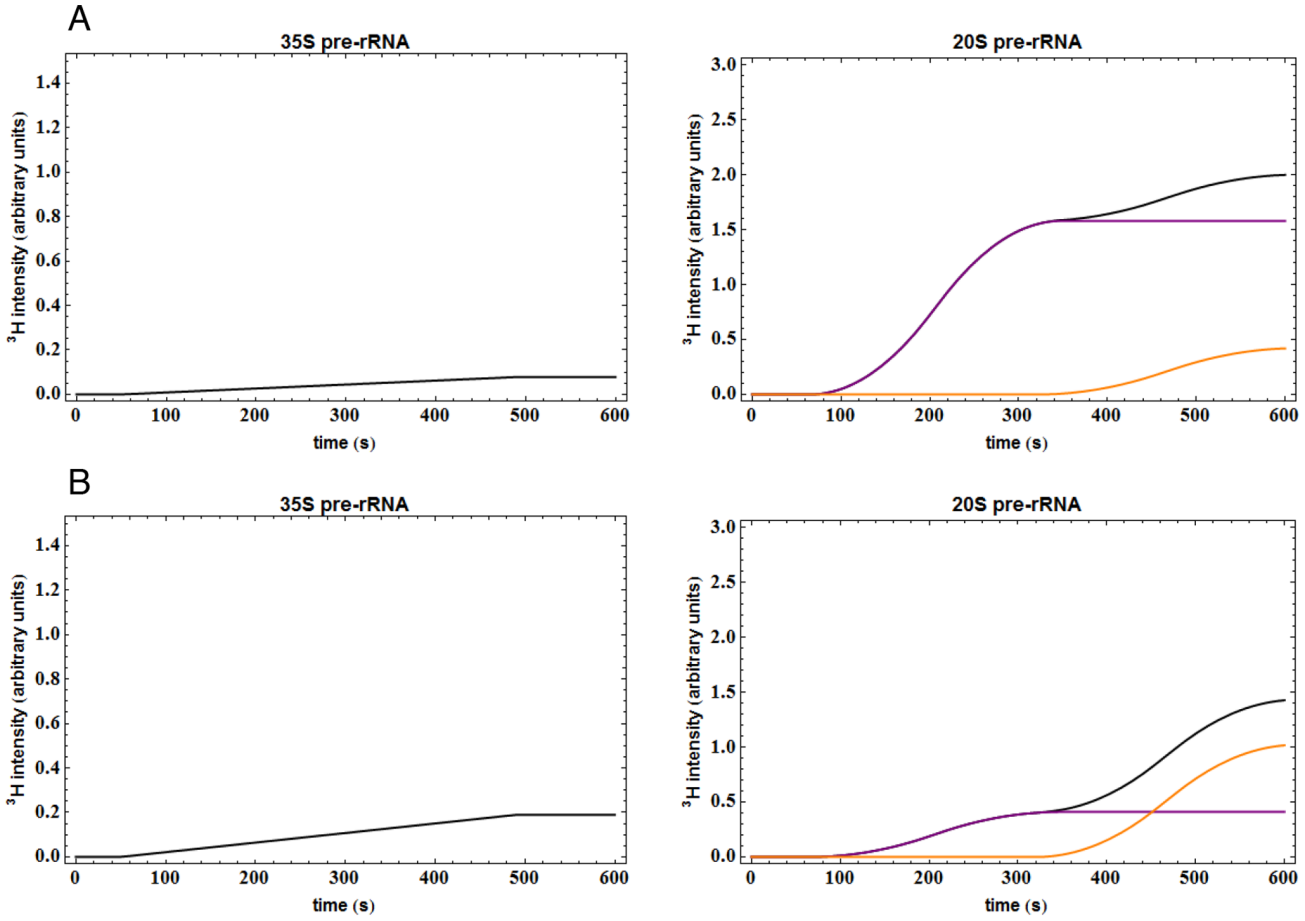
Comparison of predicted labeling curves with different levels of cotranscriptional pre-rRNA cleavage. (A) Modeled incorporation into 35S and 20S pre-rRNA with high (70%) cotranscriptional cleavage. (B) Modeled incorporation into 35S and 20S pre-rRNA with low (30%) cotranscriptional cleavage. Purple lines represent the NTC population. Orange lines represent the RTC population. The black line is the sum of the NTC and RTC populations.

To aid data analysis two software tools, M Fit and S Fit, were. M Fit is a tool for visualizing pre-rRNA processing network dependencies. In Figure 2, M Fit was used to predict the effects of alterations in the NTC : RTC ratio on the time courses of label incorporation into different pre-rRNA species. S Fit was used to investigate the possibility of automatically determining pre-rRNA lifetimes and other parameters from mathematical models populated with the data derived from metabolic labeling experiments. We could show that using only the labeling data, unique solutions to the equations cannot be obtained, since the data for 35S transcription time, lifetime and frequency of NTC are inter-connected (Figure S1). If one of these parameters is fixed then reliable values for other pre-rRNA parameters can be obtained. However, setting this value must be done using independent data that is not derived from the experiment. As an example, in Figure S2 the probability (P) of NTC has been set to 70%. The velocity of transcription (V) can be determined without recourse to modeling by considering the time course of labeling. At early time points all newly synthesized pre-rRNAs are incompletely labeled, with a 5′ domain of unlabeled RNA and a fully labeled 3′ domain. During the time course of labeling, the unlabeled region becomes smaller, until the entire length of the pre-rRNA is labeled, at which point a plateau, or steady state is reached. The transcription time of 35S synthesis can therefore be reliably determined from the time required to reach steady state - less the label equilibration time and the 35S life-time. The lifetime of 35S was previously reported to be ∼10 sec from steady-state analyses. The equilibration time can be determined by analysis of labeling of the 5S rRNA, which is robustly transcribed and readily detected, but requires only ∼3 sec for transcription, due to its small size. Analysis of incorporation into 5S, indicated that 45 sec was required for equilibration. Inspection of rRNA genes in “Miller” chromatin spreads by EM [6] and modeling of the kinetics of in vivo labeling of rRNA [7] each indicated around 70% of nascent pre-rRNAs undergo NTC in wild-type cells.

The revised model was used to determine the predicted changes in labeling kinetics for yeast strains with wild-type (70%) or reduced (30%) levels of NTC (Figure 2). The parameters used (listed in Table 2) are based on published values [7] but were modified to show more clearly the features of the labeling curves. The probability of NTC (P) was the only value altered between the curves. For 35S, reduced NTC did not alter the delay before incorporation. However, the slope of the line and the height of the plateau in 35S incorporation were elevated (Figure 2B). This reflects the increased probability that the transcribing polymerase will synthesize the full-length transcript, resulting in an increased flux through the 35S pre-rRNA on the RTC pathway. In the case of 20S pre-rRNA, the delay before incorporation, the rate of incorporation and the height of the plateau are all sensitive to the NTC:RTC ratio. To make the curves more explicit, the predicted contributions of the NTC and RTC pathways to 20S labeling have been separated in Figure 2. In the NTC pathway (purple lines), labeled 20S appears as soon as the polymerase has reached a “trigger point” for cleavage [7], which is located approximately 1.5 kb 3′ to site A2. In contrast, the appearance of labeled 20S in the RTC pathway (orange lines) requires the polymerase to pass cleavage site B0 at the 3′ end of the 35S pre-rRNA. The 35S can then be released and processed to 20S and 27SA2 via the RTC pathway. The additional time required for transcription between the trigger point for NTC and site B0 for RTC (∼70 sec), plus the lifetime of 35S (∼10 sec) are responsible for the kinetic delay in 20S labeling on the RTC pathway relative to NTC. The overall 20S labeling curve is therefore displaced to the right as a consequence of an increased RTC to NTC ratio.

### Application of Kinetic Labeling to Strains Depleted of Rat1

**Figure 3:**
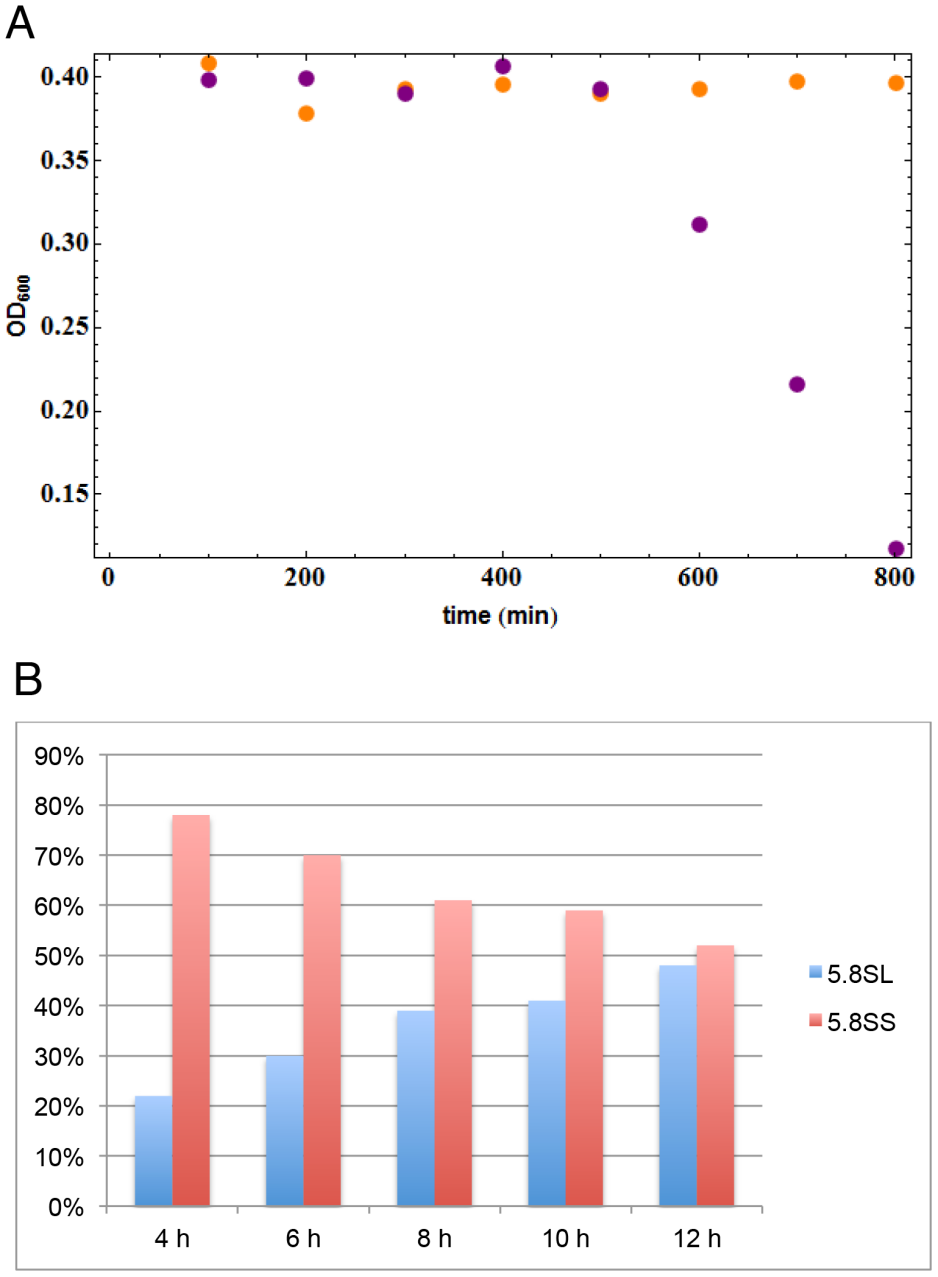
Time courses of phenotypes in strains depleted for Rat1. (A) OD_600_ of the cultures at the time points indicated. Non-depleted cells, growing in the absence of methionine, were maintained in exponential growth phase by frequent dilution with pre-warmed medium. Rat1-depleted cells, growing in the presence of methionine, were diluted at the same times and to the same extent as non-depleted cells. OD_600_ values (cell density) for the non-depleted strain at the time points indicated are shown in orange. OD_600_ values for the Rat1-depleted strain at the same time points are indicated in purple. Doubling time for the non-depleted strain was ∼100 min. (B) Relative abundances of 5.8S(L) (Rat1-independent) and 5.8S(S) (Rat1-dependent), with total 5.8S rRNA abundance set to 100% at each time point. Graphs show the averages of three independent experiments.

Modeling indicated that metabolic labeling should readily detect the time delay caused by mutations that impair NTC. To assess whether depletion of Rat1 results in NTC inhibition, the chromosomal *RAT1* gene was placed under the control of a repressible *MET3* promoter [19]. The *P_MET3_::RAT1* strain was transformed with a low copy number *CEN* plasmid expressing HA-tagged Rat1 under the control of the *RAT1* promoter, or with the empty vector. In addition, all strains were transformed with a *URA3* plasmid to allow pre-growth of the strains on medium lacking uracil prior to labeling with [^3^H-5,6] uracil. Following methionine addition, growth of the *P_MET3_::RAT1* strain was progressively impaired, commencing 10 h after methionine addition (Figure 3A). Rat1 is required for normal 5′ maturation of the major, short form of 5.8S rRNA (5.8S(S)), but is not required for the alternative, long form (5.8S(L)). Northern analyses confirmed the replacement of 5.8S(S) rRNA with 5.8S(L) during Rat1 depletion (Figure 3B). Expression of HA-tagged Rat1 from the plasmid was confirmed by western blotting (data not shown). Following Rat1 depletion for 8 h, 5.8S processing was altered but growth was not clearly impaired, and functional analyses were therefore performed at this time point.

Following addition of methionine for 8 h, Rat1 depleted strains and non-depleted cells were pulse-labeled with [^3^H-5,6] uracil. Cells were harvested at 30 sec intervals, RNA was extracted, separated on gels, and visualized using a Fuji imager. Figure S3 shows a representative image of the separation of labeled RNA. To allow different data sets to be directly compared, signals were normalized to the average values for the 27SA pre-rRNA plateau, which was previously shown to give the most reliable results [7]. To correct for differences in extraction and RNA recovery, filters were hybridized with [^32^P] labeled probes directed against the mature 18S and 25S rRNAs following analysis of the [^3^H] labeling data.

**Figure 4:**
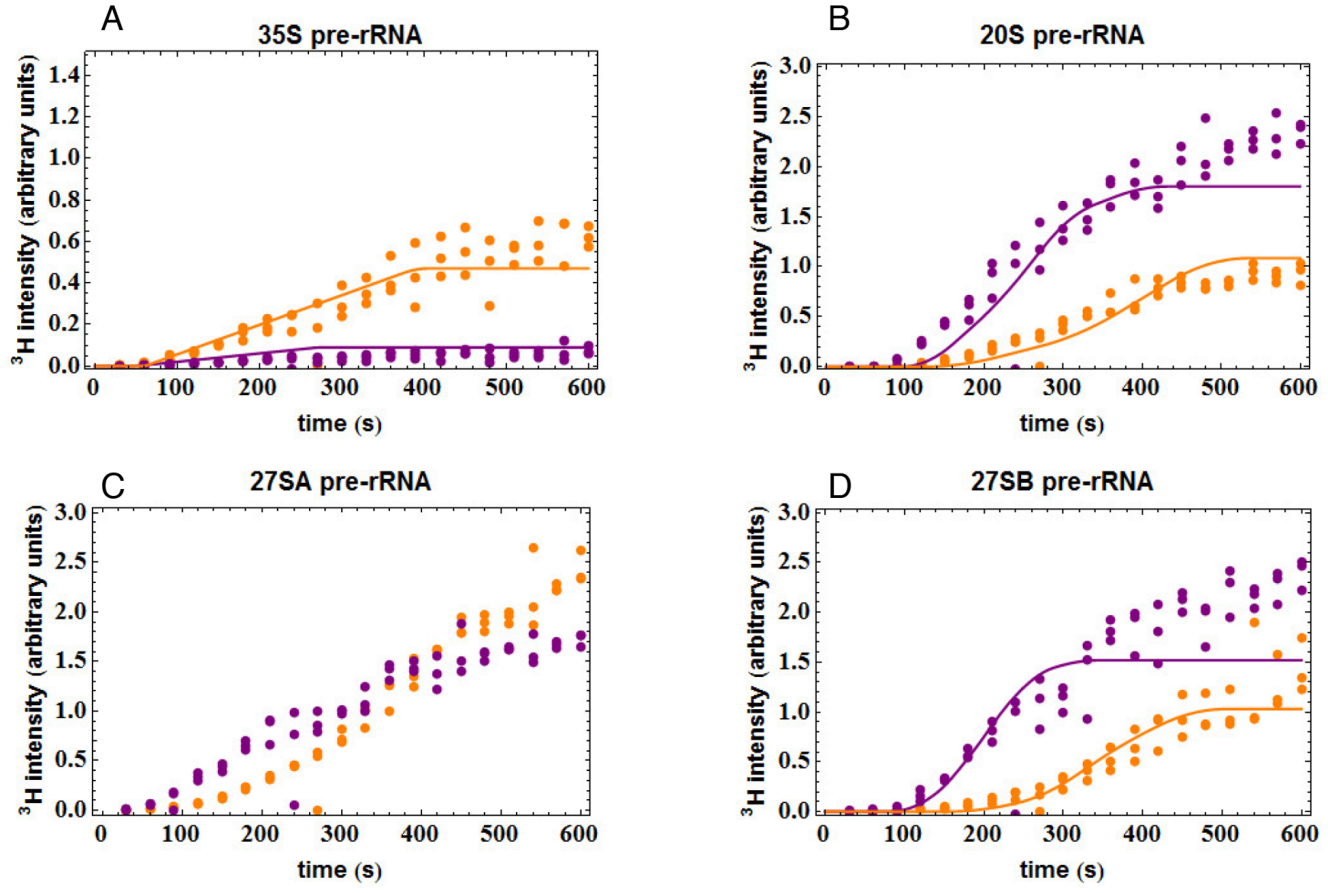
Depletion of Rat1 inhibits NTC. Kinetics of pre-rRNA labeling in *P_MET3_::RAT1* strains carrying the plasmid expressing Rat1 (shown in purple) or the empty plasmid (shown in orange), following growth in the presence of methionine for 8 h to repress expression of chromosomal Rat1. (A) Incorporation into 35S pre-rRNA. (B) Incorporation into 20S pre-rRNA. (C) Incorporation into 27SA pre-rRNA. (D) Incorporation into 27SB pre-rRNA. The three points shown for each time point represent the values obtained from three independent experiments. Solid lines represent the modeled response using the values from Table 3. Values for Rat1-expression are shown in purple and values for Rat1-depletion in orange.

The labeling curve for 35S pre-rRNA was considerably elevated in the Rat1 depleted strain (yellow points in Figure 4A). This would be consistent with increased 35S pre-rRNA synthesis in the Rat1 depleted strain due to less frequent co-transcriptional cleavage. Also consistent with reduced NTC, the 20S pre-rRNA signal was reduced and delayed following Rat1 depletion (Figure 4B). The 27SA pre-rRNA was used for normalization, so the experimental data cannot usefully be analyzed (Figure 4C).

Not all changes observed in the depletion strain can be attributed to reduced NTC. Rat1 functions directly as an exonuclease in trimming from site A3 to site B1(S), the 5′ end of the major, 27SBS pre-rRNA and the mature 5.8S(S) rRNA [1]–[3]. In consequence, metabolic labeling of 27SB showed a delay and lower plateau (Figure 4D), reflecting the inhibition of 27SA to 27SB processing expected in the Rat1 depleted strain. However, overall accumulation of mature 5.8S was not strongly impaired by Rat1 depletion, due to additional exonucleases Rrp17 and Xrn1 that process 5.8S(S) plus the existence of an alternative pathway that generates the 5.8S(L) rRNA [3], [5], [19], [20].

We next compared the experimental data to the model (solid lines in Figure 4). Parameters used for modeling are listed in Table 3. For the Rat1 expressing strain the initial values were based on previous modeling data [7], with the probability (P) of NTC set to 70%. In contrast, P was set to 30% for the Rat1 depleted strain. The value of P (probability of NTC) was the most significant factor influencing the fit of the model to the experimental data (see Figure S4, in which only the value for P was altered), however, modification of other parameters was also required. In the Rat1 expressing strain the lifetime of 35S was determined as 9 sec, in good agreement with previous estimates (see [7]), but was increased to 30 sec following Rat1 depletion, possibly as a consequence of the increased flux through this pathway. In addition, the modeled transcription elongation rate was decreased from 30 nt sec^−1^, to 20 nt sec^−1^ during Rat1 depletion. The *in vivo* transcription rate for RNA pol I was previously reported to be 60 nt sec^−1^, based indirectly on the overall rate of ribosome synthesis and the number of transcribing polymerases [18], whereas previous modeling data determined the transcription rate of a different wild-type strain to be 40 nt sec^−1^.

The other major changes following Rat1 depletion were the increases in the 27SA lifetime and the time for 27SA to 27SB processing (Table 3), reflecting the direct involvement of Rat1 activity in processing the major 5′ end of 27SB pre-rRNA.

The effects of Rat1 depletion are in good overall agreement with the consequences predicted for inhibition of NTC. There are, however, some differences from the theoretical kinetics shown in Figure 2, which presents an optimal case where only co-transcriptional probability was changed and the other parameters were adjusted to display the features of the curves. The kinetics in Figure 4 used parameters that gave an overall best fit to the actual values for the Rat1 expressing and depleted strains. In addition to P, which had the major effect, this involved changes in transcription speed and lifetime of 35S. In Figure 2a marked, late inflection is visible as RTC starts to contribute to the observed 20S pre-rRNA labeling. This increase is present in Figure 4B, but forms a shoulder rather than a discrete inflection. In contrast, the late increase in 20S labeling in the experimental data in Figure 4 is likely to arise from the behavior of the uracil pumps leading to increased uptake at later time points, possibly coupled to a decrease in endogenous uracil synthesis. There are multiple uracil pumps with differing affinities that are subject to complex regulation. This model is consistent with an increase observed in labeling of the 5S rRNA, for which the transcription time is very short and little processing occurs (data not shown).

### Expression of Catalytically Inactive Rat1_D235A_

**Figure 5:**
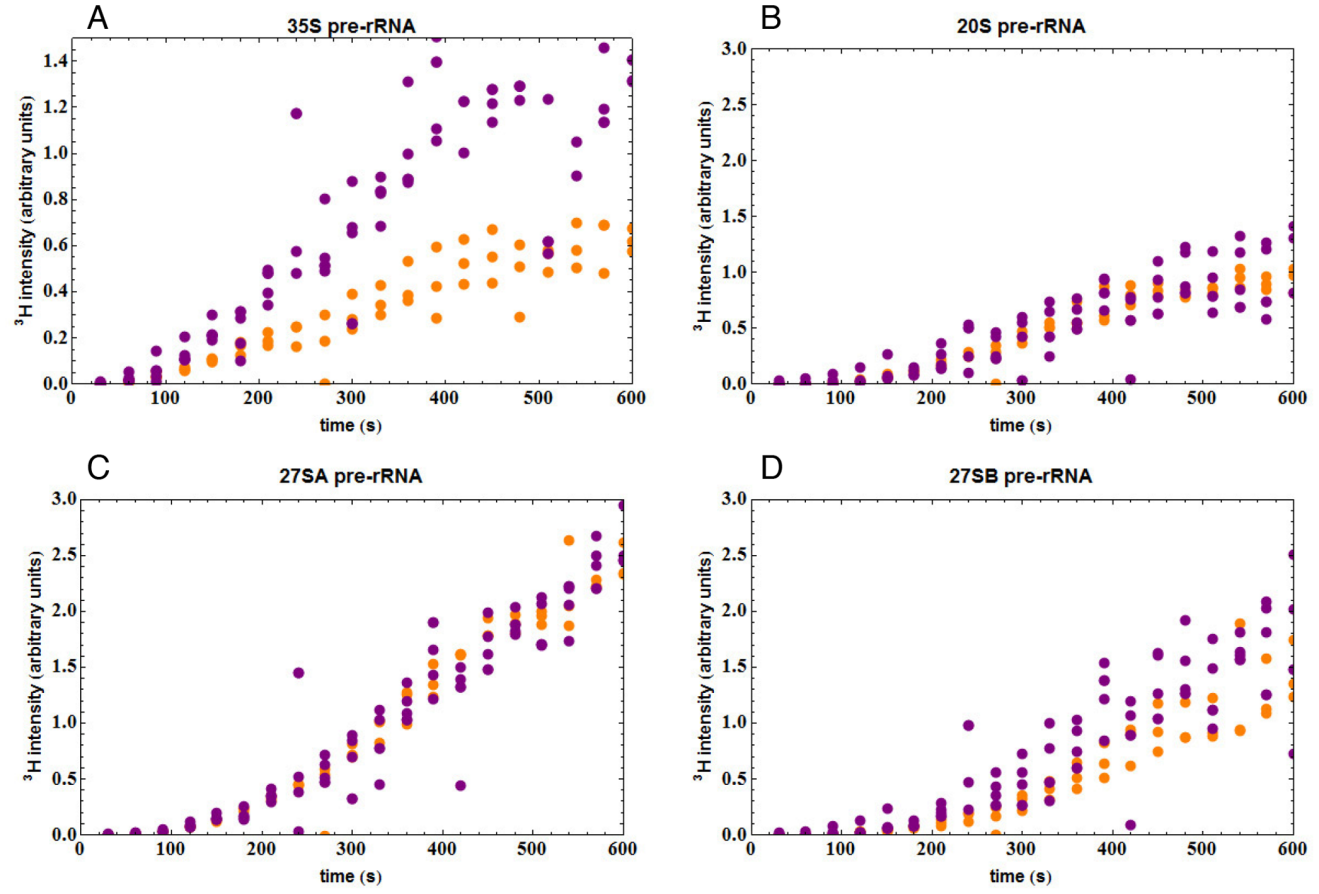
Expression of catalytically inactive Rat1_D235A_ increases 35S pre-rRNA accumulation. Kinetics of pre-rRNA labeling in *P_MET_::RAT1* strains carrying the empty plasmid (shown in orange) or the plasmid expressing Rat1_D235A_ (shown in purple) following growth in the presence of methionine for 8h to repress expression of chromosomal Rat1. (A) Incorporation into 35S pre-rRNA. A higher plateau for 35S was observed in the strain expressing Rat1_D235A_. (B–D) Incorporation into 20S, 27SA and 27SB pre-rRNAs, respectively. Differences in labeling kinetics for 20S, 27SA and 27SB pre-rRNAs were not significant (p>0.05) as shown by a student’s T-test. The T-test determines the probability that two samples come from the same population, and was performed based on a two-tailed distribution.

The analyses presented above indicated that depletion of Rat1 resulted in reduced NTC. A D235A point mutation in Rat1 has been well characterized, and shown to block catalytic activity [19], [21]. In an attempt to assess whether the exonuclease activity of Rat1 was required for NTC, Rat1_D235A_ was expressed from a plasmid in the *P_MET3_::RAT1* strain. During growth in the absence of methionine, the presence of the plasmid expressing Rat1_D235A_ conferred no clear defect in growth (data not shown) or pre-rRNA processing (Figure S5). This indicates that the catalytically inactive Rat1_D235A_ protein is not dominant negative over wild-type Rat1. When methionine was added to the medium to repress the synthesis of wild-type Rat1, expression of Rat1_D235A_ resulted in a strong increase in accumulation of the 35S pre-rRNA compared to the strain that was only depleted of Rat1 (Figure 5A), indicative of a further reduction in NTC. Clear changes were not seen for the 20S pre-rRNA, but the very short lifetime of the 35S pre-rRNA makes it much more sensitive to the effects of mild delays in processing. Moreover, Rat1 has been reported to degrade the 35S pre-rRNA [22], so it is possible that the 35S accumulation in the absence of Rat1 activity is independent of processing defects.

### Miller Chromatin Spreads Confirm Decreased Co-transcriptional Cleavage

Analyses of chromatin spreads can provide insights into the transcription and processing of single rDNA genes. The nascent pre-RNA transcripts give Miller spreads their “Christmas Tree” like appearance. The prominent terminal balls decorating the transcripts are pre-40S complexes, called SSU processomes, which contain the 5′ regions of the nascent transcripts packaged together with proteins and snoRNA [15], [16]. Loss of these terminal balls in the 3′ region of the rDNA identifies those pre-rRNA transcripts that have undergone NTC.

The requirement for Rat1 in NTC was independently assessed by comparison of *P_MET_::RAT1* strains carrying the plasmids expressing Rat1, the empty plasmid or the catalytically inactive Rat1_D235A_.

**Figure 6:**
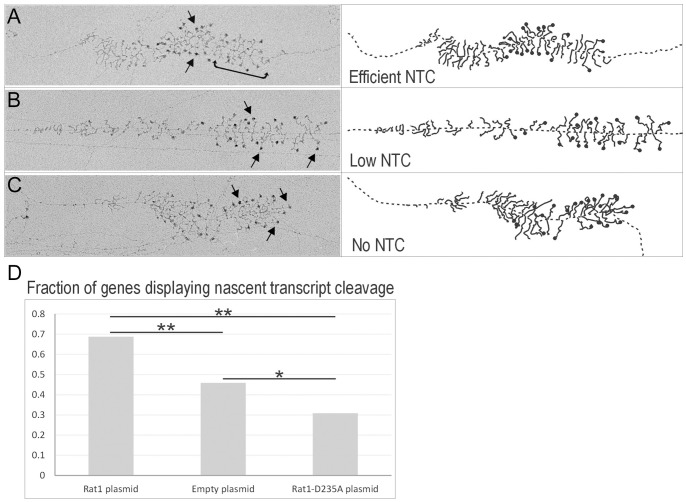
Miller chromatin spreads of rDNA from Rat1 depletion strains. (A–C; left) Representative EM images showing a single rDNA gene with typical efficient NTC (A), low NTC (B), and no NTC (C). Genes are orientated with the direction of transcription from left to right. Arrows indicate examples of SSU processomes, and the bracket indicates a gene region with mostly cleaved transcripts. (A–C; right) Simplified cartoon versions of the EM images. The branching structures visible in the images are nascent pre-rRNA transcripts, emanating from the central rDNA strand. The black balls are SSU processome complexes, which assemble cotranscriptionally and compact the 5′ regions of the nascent transcript. The balls are lost from pre-rRNA transcripts at the 3′ end of the rDNA (on right of images) that have undergone NTC, as best shown in panel A. (D) Fraction of genes showing co-transcriptional cleavage. More than 75 genes were analyzed per yeast strain. In the presence of methionine, the fraction of genes with NTC decreased significantly in the empty plasmid strain over the Rat1 plasmid strain (P = 1.4×10^−6^), with further reduction in the Rat1_D235A_ strain (Rat1_D235A_ to empty plasmid P = 0.008; Rat1_D235A_ to Rat1 wild-type P = 5.1×10^−13^).

In cells expressing Rat1, the SSU processomes are largely lost from nascent transcripts about two thirds of the way into the rDNA gene, due to A0-A2 cleavage and release of the pre-40S particles (Figure 6A) [6]. In the strains depleted of Rat1 or expressing only catalytically inactive Rat1_D235A_, a higher proportion of genes retained the terminal balls on most or all of the nascent transcripts, demonstrating reduced NTC. Representative examples are shown in Figs. 6B and C, respectively. Notably, formation of the terminal balls was not clearly affected by Rat1 depletion or expression of Rat1_D235A_, indicating that assembly of the SSU processome complex was not inhibited.

Quantification of the data from multiple individual rDNA genes (>75 for each strain) (Figure 6D) confirmed that the fraction of genes exhibiting NTC was decreased in the Rat1-depleted strain and further decreased by expression of Rat1_D235A._ This decrease in cleaved transcripts seen by EM analysis is in agreement with the increase in 35S pre-rRNA seen by metabolic labeling in these strains (Figure 5A). However, Rat1_D235A_ was not clearly dominant negative for NTC in the absence of methionine (non-depleted conditions), consistent with the metabolic labeling (Figure S5, and data not shown).

Together these data show that yeast strains depleted of Rat1 show decreased cotranscriptional pre-rRNA cleavage.

## Discussion

Here we have used kinetic analyses and mathematical modeling to test the hypothesis that depletion of the 60S ribosome subunit synthesis factor Rat1 inhibits the early cotranscriptional cleavage steps in pre-40S rRNA processing, in addition to its known targets in 60S subunit maturation.

The products of cleavage of the nascent and released pre-rRNAs are not readily distinguished at steady-state, necessitating kinetic analyses. We initially aimed to produce a model that would allow the processing parameters to be directly and automatically calculated from kinetic data on the incorporation of metabolic label into the pre-rRNA and rRNA species. To predict the consequences of the inhibition of NTC on the kinetics of pre-rRNA labeling, we rewrote our MS Excel-based algebraic model in the Mathematica 6.0 programming language, making it more transferable and flexible. In addition two applications were written, designated M-Fit and S-Fit. M-Fit allows visualization of the pre-rRNA processing network and changes that result from perturbations. Experimental data can be loaded in the background and manual parameter fitting can be performed. The S-Fit program can be used to automate the fitting process and find best fit values for model parameters.

When used without constraints, the parameter values returned by S-Fit were ambiguous, due to the parameter interrelations in the models. Indeed, further analyses demonstrated unambiguously that a unique best fit cannot be obtained using only the kinetic data. To achieve useful fits, it was necessary to define values and limits for specific parameters. Suitable parameter values are the lifetime for 35S pre-rRNA or the transcription elongation rate. Defining the lifetime of 35S based on published values allowed a good parameter fit for the probability of co-transcriptional cleavage.

Problems with inter-related data are expected to be common when modeling data obtained from biological experiments. For example, mRNA synthesis and turnover rates are related. Their determination from metabolic labeling using 4-thiouridine therefore required the inclusion of constraints on the modeling [23]. In this case the authors included the assumption that the mRNA lifetimes are generally much longer than the 6 min labeling time, based on prior, independent data.

To experimentally determine the requirements for Rat1 in NTC, the endogenous *RAT1* gene was placed under the control of a repressible *MET3* promoter, allowing its depletion by addition of methionine to the growth medium. The labeling kinetics observed experimentally in strains depleted of Rat1 were in close agreement with the predicted effects of NTC inhibition derived from the mathematical model. This was also in good agreement with the level of residual NTC in the Rat1 depleted strain determined from EM imaging.

Pre-rRNA processing at sites A0-A2 releases the 20S pre-rRNA and is entirely endonucleolytic, whereas Rat1 has only 5′–3′ exonuclease activity. Despite this we wanted to determine whether the nuclease activity of Rat1 was required for NTC. Comparison of the effects of Rat1 depletion with its replacement by catalytically inactive Rat1_D235A_ [21] showed a substantially higher plateau for 35S in the strain expressing Rat1_D235A_. This indicates that the catalytically inactive Rat1 protein has a negative effect, which actively delays 35S processing. It is notable that the catalytically inactive Rat1_D235A_ protein was not “dominant negative”, since it conferred no clear phenotype in the presence of normal levels of functional Rat1. It did, however, exacerbate the effects of Rat1 depletion. This “recessive negative” phenotype might be indirect. Possible explanations include the accumulation of Rat1_D235A_ in complexes with substrate RNAs, leading to the sequestration of Rat1-associated proteins in non-productive complexes (e.g. the Rat1 cofactor Rai1) [24], [25]. Alternatively, recruitment of the inactive Rat1 to pre-rRNA might conceivably block the action of the Rrp17 5-exonuclease, which is partially redundant with Rat1 for pre-rRNA processing and shows an early processing defect that is similar to Rat1 [5]. It is also notable that Rat1 participates in degradation of the 35S pre-rRNA in cells without other processing defects [22]. Recent data indicate that newly synthesized RNAs transcribed by RNA Polymerases II and III undergo a very substantial level of nuclear degradation [26], [27]. This may also be the case for the Pol I transcribed 35S pre-rRNA, so the catalytically inactive Rat1 might lead to the stabilization of pre-rRNA species that would otherwise have been degraded.

To independently assess the effects of depletion of Rat1 on the NTC pathway, EM images of “Miller” chromatin spreads of rDNA genes were also analyzed. This confirmed the decreased probability of NTC in strains depleted of Rat1. Consistent with the modeling data, NTC was not abolished in the *P_MET3_::RAT1* strain. However, depletion of Rat1 is not expected to be complete, since the analyses were performed after only 5 h of depletion and prior to the appearance of growth defects, in order to minimize indirect effects.

Proteomic analyses confirm that Rat1 is present in pre-ribosomes prior to its function in exonuclease processing from site A3 [17]. It is therefore feasible that prior to co-transcriptional cleavage, the SSU processome “verifies” that factors that act immediately downstream in early pre-60S maturation are associated with the assembling pre-ribosomes. Although the cleavage at site A2 takes place on the nascent transcript, cleavage does not immediately follow transcription of the cleavage site. The modeling data and EM imaging both indicate that the transcribing polymerase travels around 1.5kb past site A2 before the associated pre-rRNA is cleaved. It seems plausible that during the time required for this transcription (∼35sec) early-binding 60S synthesis factors, including Rat1, can associate with the nascent pre-rRNA and promote cotranscriptional cleavage. Conceivably, this association might have a proofreading function. The presence of the correctly assembled, early 60S processing factors would indicate that general pre-ribosome assembly was progressing correctly. This might then present a “ready for processing” signal to the nuclease(s) responsible for A0-A2 cleavage.

## Supporting Information

Figure S1 **Surface graph fitting without fixed parameters.** These graphics show the relationship between the parameters in the 35S model when performing automatic fitting. Both curves present the same information from different perspectives. The three axes are; P, which represents the probability of NTC in percentage; lifetime 35S, which is the lifetime of the 35S pre-rRNA in seconds; and fitting error in arbitrary units. A good fit is represented by a valley on the graph.
(TIF)

Figure S2 **Surface graph with P fixed.** The figure shows a surface graph of the 35S model where P (probability of co-transcriptional cleavage) is fixed at 70% NTC. Here the three axes are; lifetime of 35S, the fitting error in arbitrary units and the time-course of the kinetic analysis. A clear valley for the lifetime 35S parameter at circa 15 sec is shown. Hence the lifetime 35S parameter would have a value of 15 sec, as determined by best-fit search.
(TIF)

Figure S3 **Time course of labeling.** Representative gel showing the time course of label incorporation into the pre-rRNA and rRNA species indicated on the left. The RNAs were separated on an agarose glyoxal gel, transferred to Hybond N+ membrane and visualized by scanning of the membrane with a Fuji scanner.
(TIF)

Figure S4 **Alteration of only the probability (P) of NTC has a major effect on data fitting.** The parameters for Rat1-expression are as in Figure 4. For Rat1-depletion only the value of P (the probability of NTC) was altered from 70% to 30%.
(TIF)

Figure S5 **Expression of catalytically inactive Rat1_D235A_ is not dominant negative for pre-rRNA processing.** Kinetics of pre-rRNA labeling in *P_MET_::RAT1* strains carrying the empty plasmid (shown in orange) or the plasmid expressing Rat1_D235A_ (shown in purple) during growth in the absence of methionine to allow expression of chromosomal Rat1. There are no significant differences between the two sets of samples.
(TIF)
