# Supplementary figures and images for: Kinetic Analysis Demonstrates a Requirement for the Rat1 Exonuclease in Cotranscriptional Pre-rRNA Cleavage
Source: PLoS One. 2014 Feb 3;9(2):e85703. doi: 10.1371/journal.pone.0085703 (PMC3911906; doi:10.1371/journal.pone.0085703)

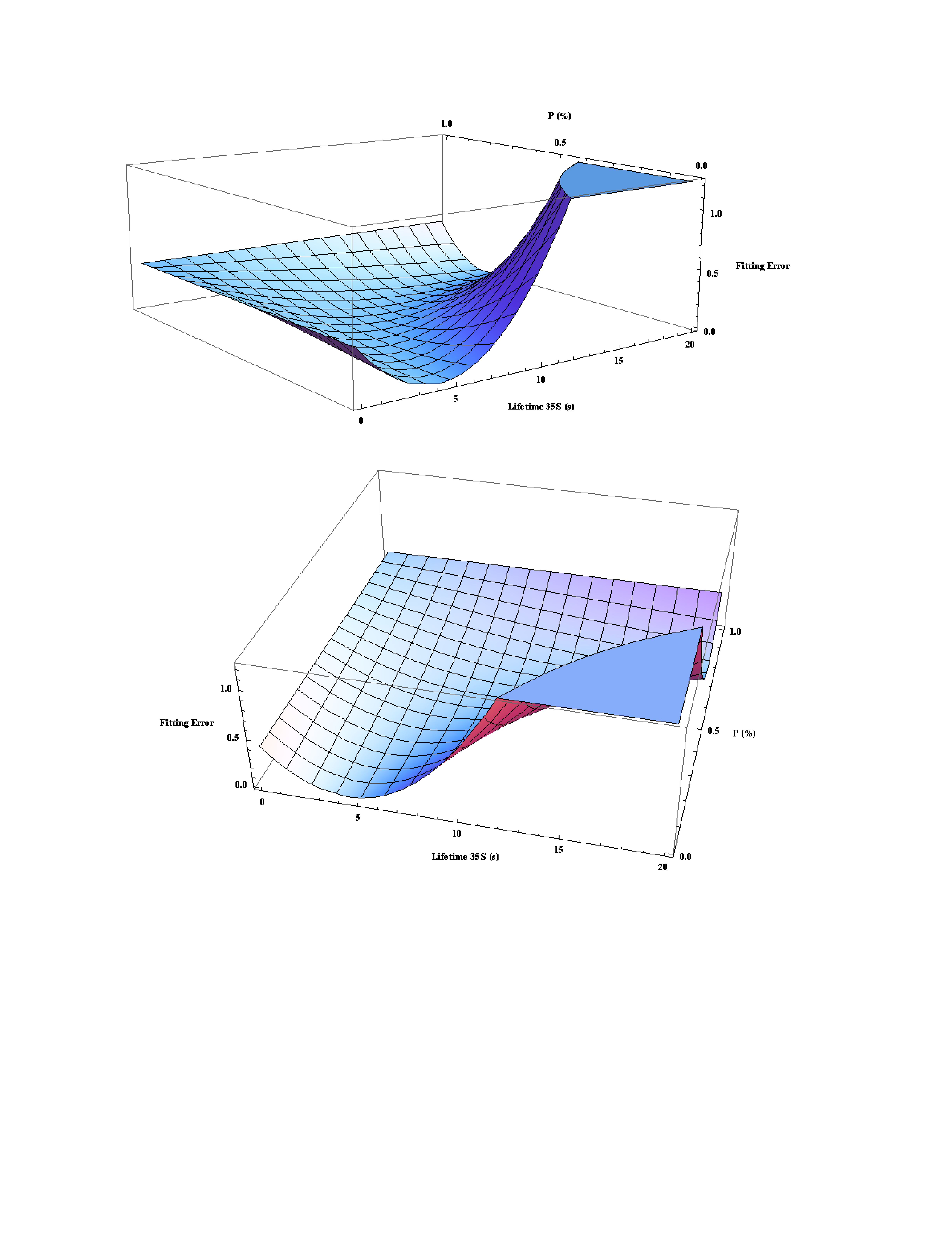

Supplement: Figure S1 — Surface graph fitting without fixed parameters. These graphics show the relationship between the parameters in the 35S model when performing automatic fitting. Both curves present the same information from different perspectives. The three axes are; P, which represents the probability of NTC in percentage; lifetime 35S, which is the lifetime of the 35S pre-rRNA in seconds; and fitting error in arbitrary units. A good fit is represented by a valley on the graph. (TIF) [file pone.0085703.s001.tif]

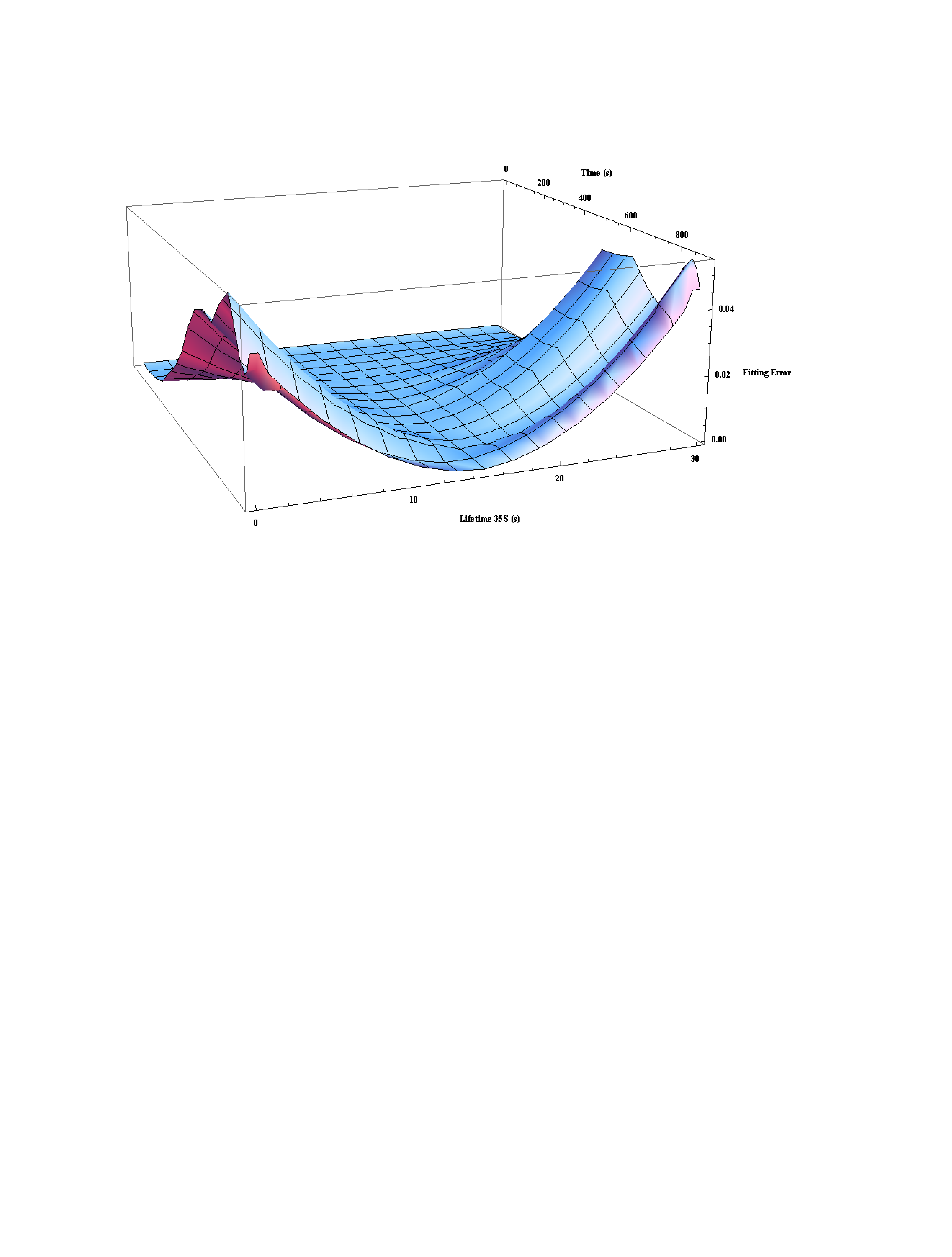

Supplement: Figure S2 — Surface graph with P fixed. The figure shows a surface graph of the 35S model where P (probability of co-transcriptional cleavage) is fixed at 70% NTC. Here the three axes are; lifetime of 35S, the fitting error in arbitrary units and the time-course of the kinetic analysis. A clear valley for the lifetime 35S parameter at circa 15 sec is shown. Hence the lifetime 35S parameter would have a value of 15 sec, as determined by best-fit search. (TIF) [file pone.0085703.s002.tif]

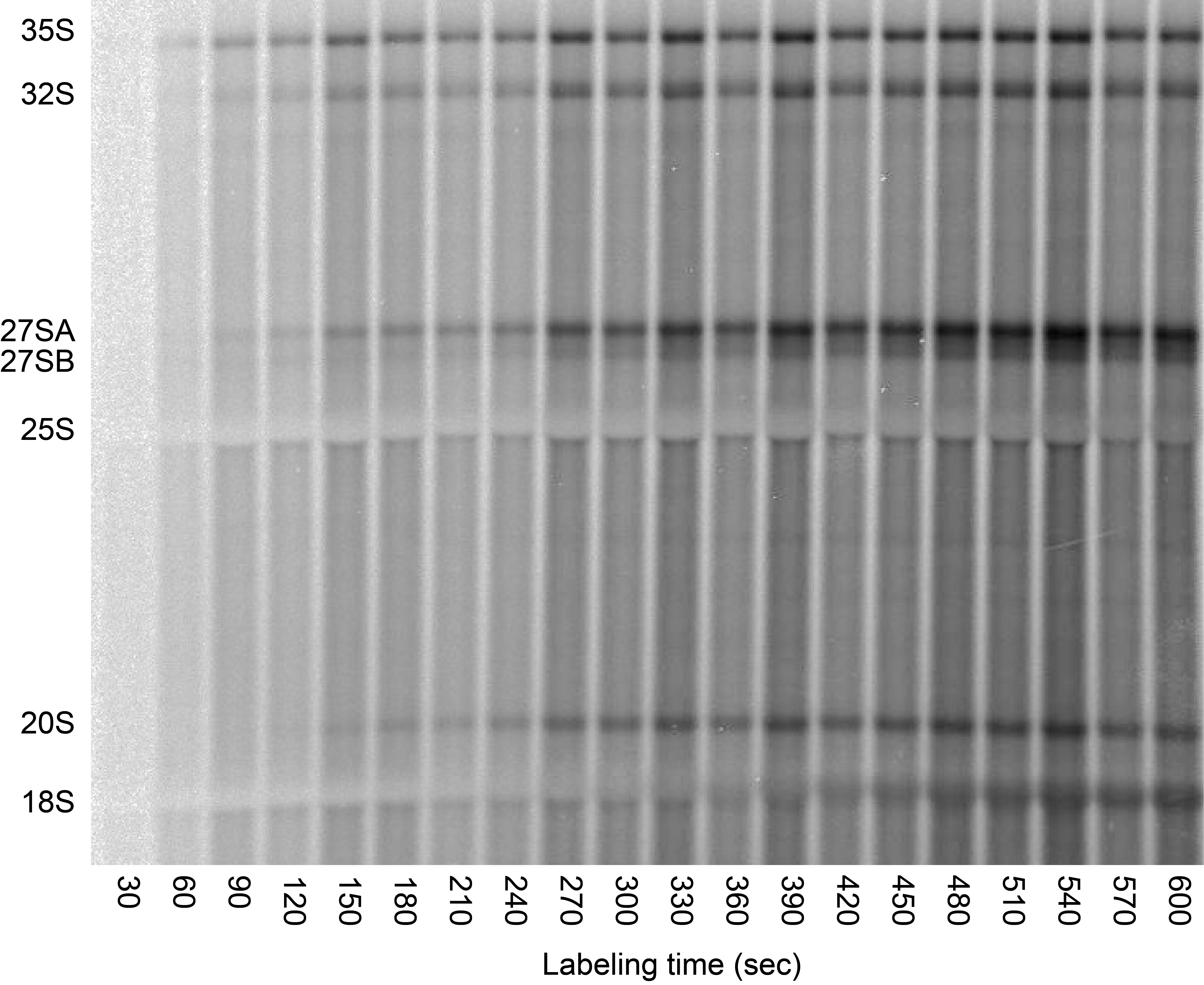

Supplement: Figure S3 — Time course of labeling. Representative gel showing the time course of label incorporation into the pre-rRNA and rRNA species indicated on the left. The RNAs were separated on an agarose glyoxal gel, transferred to Hybond N+ membrane and visualized by scanning of the membrane with a Fuji scanner. (TIF) [file pone.0085703.s003.tif]

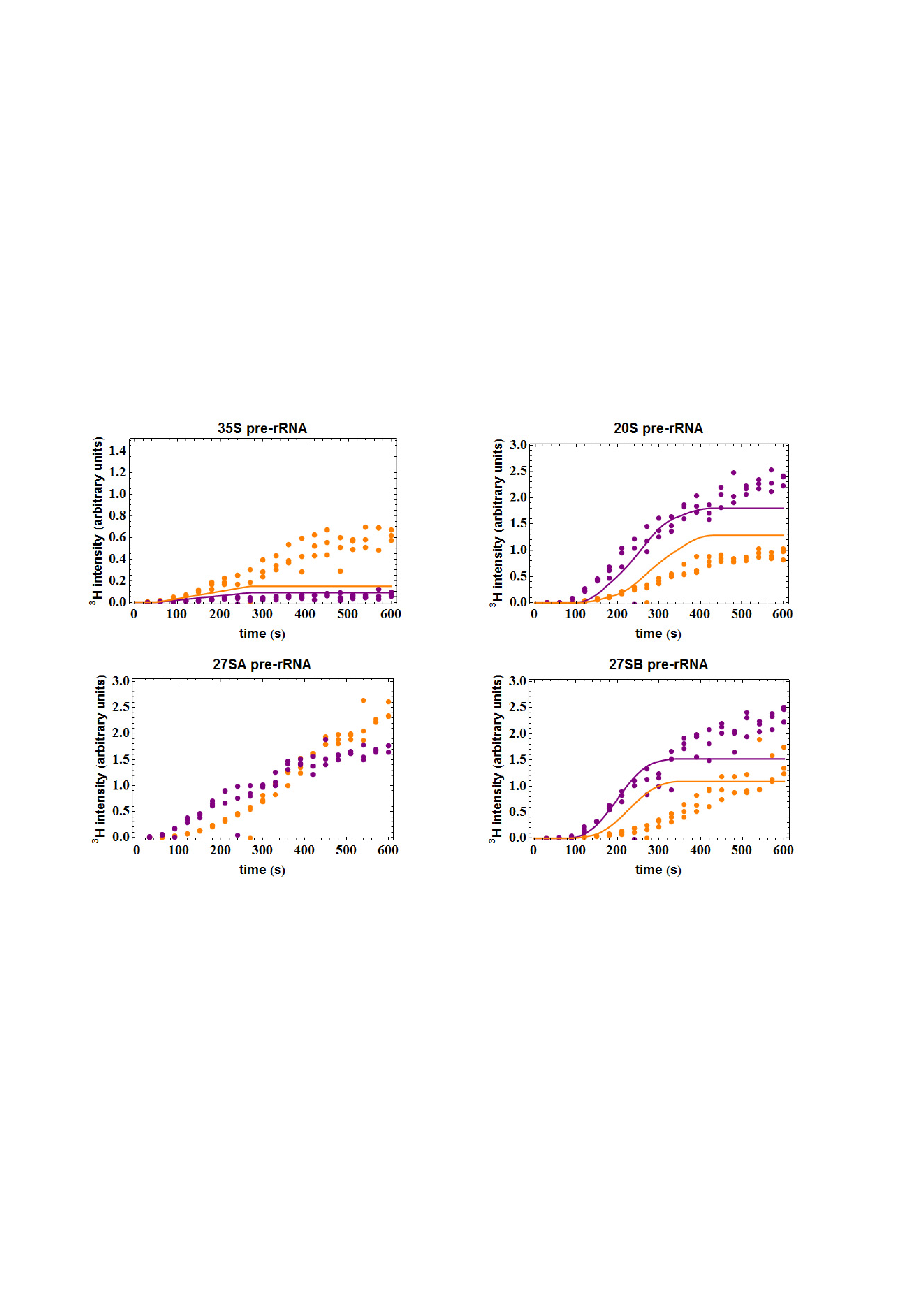

Supplement: Figure S4 — Alteration of only the probability (P) of NTC has a major effect on data fitting. The parameters for Rat1-expression are as in Figure 4. For Rat1-depletion only the value of P (the probability of NTC) was altered from 70% to 30%. (TIF) [file pone.0085703.s004.tif]

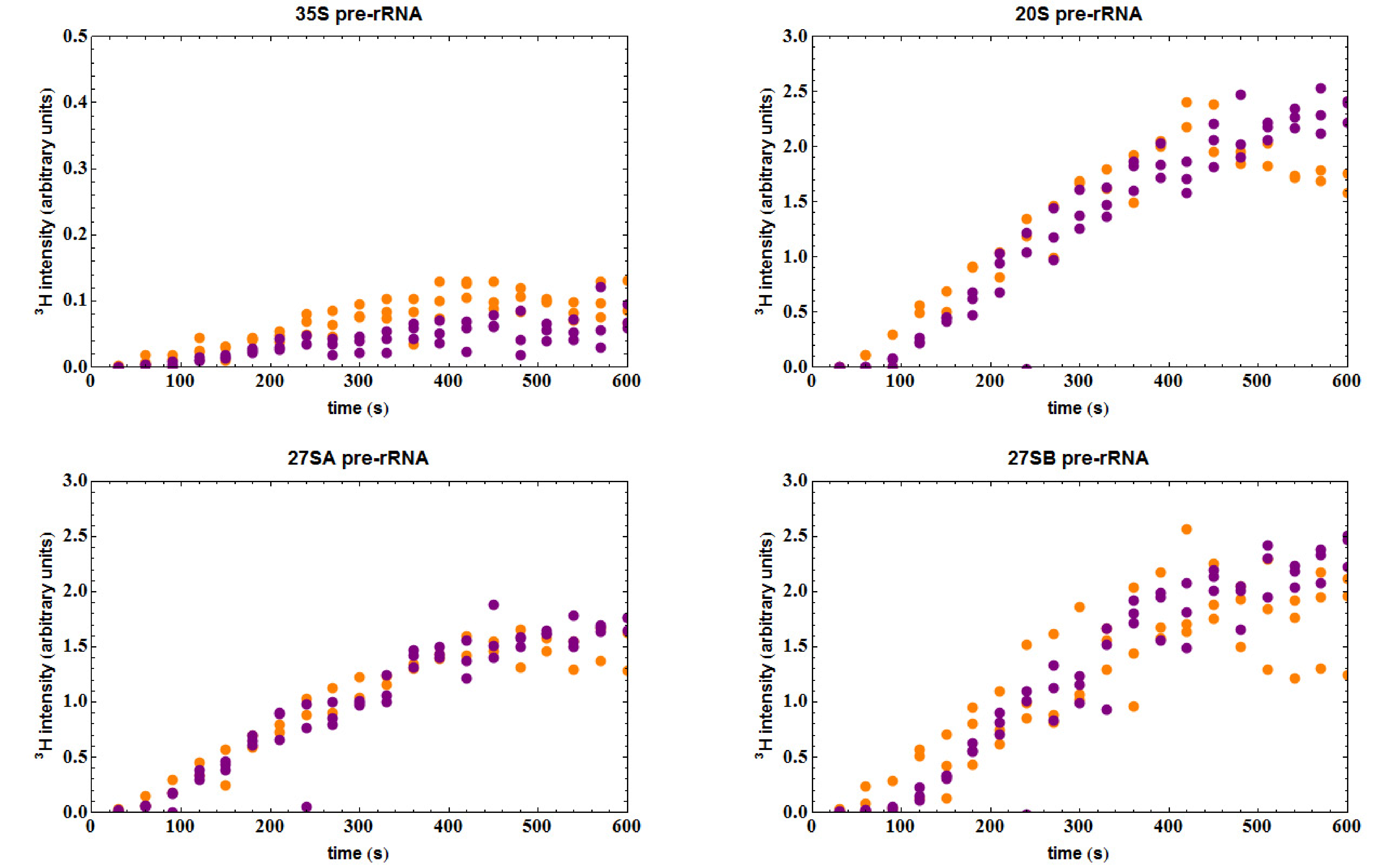

Supplement: Figure S5 — Expression of catalytically inactive Rat1D235A is not dominant negative for pre-rRNA processing. Kinetics of pre-rRNA labeling in PMET::RAT1 strains carrying the empty plasmid (shown in orange) or the plasmid expressing Rat1D235A (shown in purple) during growth in the absence of methionine to allow expression of chromosomal Rat1. There are no significant differences between the two sets of samples. (TIF) [file pone.0085703.s005.tif]
